# The “Cross Talk” between the Receptors of Insulin, Estrogen and Progesterone in Neutrophils in the Synthesis of Maspin through Nitric Oxide in Breast Cancer

**Published:** 2012-06

**Authors:** Karabi Ganguly Bhattacharjee, Mau Bhattacharyya, Umesh Chandra Halder, Pradipta Jana, Asru K. Sinha

**Affiliations:** 1 *Sinha Institute of Medical Science & Technology, Garia, 700084, India;*; 2 *Department of Chemistry, Jadavpur University, Kolkata 700032, India*

**Keywords:** breast-cancer, crosstalk, estrogen, insulin receptor, maspin, nitric oxide, neutrophils, progesterone, steroids receptors

## Abstract

**Purpose:**

The binding of either insulin, or estrogen or progesterone to their specific receptors on neutrophils has been reported to stimulate nitric oxide (NO) induced maspin synthesis in these cells. Experiments were carried out to determine the role of insulin receptor interaction in the nitric oxide induced maspin synthesis in neutrophils that was effected by estrogen or progesterone.

**Methods:**

Estrogen receptor positive (ER+) and progesterone receptor positive (PR+) neutrophils were isolated from the blood cancer subjects. Maspin was determined by enzyme linked immunosorbent assay after *in vitro* translation of maspin mRNA. NO was determined by methemoglobin method.

**Results:**

It was found that pre incubation of normal neutrophils with insulin to reach equilibrium binding decreased both ER and PR numbers by ≈50% without changing the dissociation constants of the steroids binding. The reduction of ER or PR numbers on neutrophils due to the pretreatment with insulin resulted in the decreased NO induced maspin synthesis from 2.383 ± 0.014 nM to 1.454 ± 0.004 nM in the case of estrogen and in the decrease of maspin synthesis from 2.329 ± 0.012 nM to 1.410 ± 0.002 nM in the case of progesterone. The incubation of ER+ neutrophils or PR+ neutrophils with insulin further decreased the maspin synthesis from 1.422 ± 0.029 nM to 0.790 ± 0.004 nM in the case of estrogen, and from 1.138 ± 0.024 nM to 0.555 ± 0.003 nM maspin in the case of progesterone respectively compared to normal control.

**Conclusion:**

These results suggested that a “cross-talk” between the insulin receptors and the steroid receptors down regulated maspin synthesis in normal and in breast cancer neutrophils.

## INTRODUCTION

### Background

The occupancy of receptors by its ligands (hormones) is reported to result in the activation of the receptors leading to the expression of the hormone effect in the target cells (1). The receptor ligand interaction is well known for its specificity. However it has also been reported that the occupancy of one particular hormone to its own specific receptors might up regulate or down regulate the receptors numbers of a second hormone in the same cell surface and thereby influence the expression of the effect of the second hormone (2, 3). The physiologic events that influence the effect of one ligand through its binding to its own receptors, resulted in the binding and in the consequent effect of a second ligand to its own receptors, is generally called “cross talk” between the receptors (4, 5). The increase of receptor numbers on the cell surface, which are protein macromolecules is however not always due to the synthesis of new receptor proteins in the cells and could be due to the exposure of the “spare” receptor proteins that were existed on the cell membrane bi-layers even in the absence of protein synthesis (6). We have reported before that estrogen to its binding to receptors in neutrophils led to the synthesis of maspin, an anti-breast cancer protein through the synthesis of nitric oxide (NO) (7). It was also reported that insulin, the well known anti- diabetic hormone, was also capable of stimulating maspin synthesis in neutrophils also through the synthesis of NO (8).

### Purpose

We report in the Result in this manuscript, that progesterone, like estrogen and insulin, was capable of stimulating maspin synthesis through the production of NO in neutrophils.

As insulin, estrogen and progesterone all were found to be capable of inducing maspin synthesis through NO production in neutrophils, the regulatory mechanism(s) that was involved in the synthesis of maspin through the NO synthesis, induced by these different hormones was investigated. The results of the investigation that suggested the existence of “cross talk” between the receptors of insulin with those of the steroids in neutrophils, in the context of NO induced maspin synthesis effected, by the steroids and the possible pathological implication of the crosstalk in the synthesis of the anti breast cancer protein in human breast cancer are presented herein.

## MATERIALS AND METHODS

### Ethical clearance

The protocol used in the study was approved by the Internal Review Board, Sinha Institute of Medical Science and Technology. All the participants including breast cancer patients and age matched normal female volunteers signed informed consent form. Appropriate permission was also obtained from the I.R.B. for the use of rabbits in the studies.

### Chemicals

Recombinant Human maspin (rh Maspin) was a kind gift of Dr. Sally Twining of the Dept. of Biochemistry, Medical College of Wisconsin, Milwaukee, WI, USA. The enzyme linked immunosorbant assay (ELISA) maxisorp plates were obtained from NUNC, Roskilde, Denmark. The estrogen and all other chemicals used were from Sigma Chemical Co. St. Louis, USA. ER α, ER β and PR antibodies were obtained from Thermo Fisher Scientific (Rochester, NY, USA).

### Preparation of Estrogen, Progesterone and Insulin solution

Estrogen Progesterone and Insulin solution were prepared by dissolving the compound in 0.9% NaCl, and the pH was adjusted to 7.4. The hormone solutions were immediately used after these were prepared, and discarded after use.

### Selection of patients with breast cancer

Only female breast cancer patients between 35-65 years (mean 45 years, n=20), participated in the study. The occurrence of breast cancer was diagnosed by mammogram/biopsy. None of them had received any therapy including radiation, surgery or chemotherapy but were waiting for the surgical intervention for the condition. None of the normal female volunteers and the breast cancer patients had history of diabetic mellitus, systemic hypertension, or severe infection or life threatening cardiovascular or cerebrovascular condition.

### Determination of estrogen and progesterone receptor status of neutrophils from breast cancer subjects

The isolated neutrophils were placed on glass slides, and were frozen using cold liquid nitrogen vapor, and broken by the sliding of another glass slide over them to expose the nuclear receptors in the cells to the added fluorescent antibody as reported before (7). Estrogen receptor statuses and progesterone receptor statuses were determined by immunohistochemical techniques using fluorescence tagged antibodies that recognized both α and β estrogen receptors and progesterone receptor respectively (9). The cells were immediately observed and photographed under fluorescence microscopy.

The neutrophils isolated from the peripheral blood of the breast cancer subjects were classified as estrogen receptor positive (ER+) neutrophils or estrogen receptor negative (ER–) neutrophils or progesterone receptor positive (PR+) neutrophils or progesterone receptor negative (PR–) neutrophils as described before (10). It was reported before that the statuses of receptor of either estrogen or progesterone (ER+, ER–, PR+ or PR–) in neutrophils were identical to that of the corresponding ER+, ER–, PR+ or PR– receptor statuses of the breast cancer lesion in breast cancer patients (7).

### Selection of normal volunteers

Equal number of age matched normal female volunteers with similar phases of the menstrual cycle compared to that of the selected breast cancer subjects were asked to participate in the study. These volunteers had never received any contraceptive. All volunteers were asked to stop taking any medication including aspirin for at least 2 wks before their participation in the study.

### Collection of blood

The blood samples (20-25 ml) were collected by venipuncture using siliconized 19 gauge needle in plastic vials, and anticoagulated by gently mixing 1 vol. of 0.13 M sodium citrate with 9 vol. of blood (8).

### Immunization of the animals

Polyclonal antibodies against r-human maspin, estrogen, progesterone were raised by repeated immunization in White New Zealand rabbit as described before (11).

### Assay of NO

Nitric oxide formation was assayed by methemoglobin method by following the protocol described before by using a Beckman Spectrophotometer (Model DU6) (8). The validity of the assay was confirmed by independent chemiluminescence assay of NO (12).

### Preparation of neutrophil suspension and the incubation of the isolated neutrophils with estrogen, progesterone and insulin

Neutrophils were isolated from the citrated blood samples as described before (13). The cell counts were determined by optical microscopy. The isolated neutrophils suspended in HBBS (Hanks Balanced Buffer Solution) buffer pH7.4 (6 ×10^9^ cells/L) were incubated with different concentrations of estrogen, different concentrations of progesterone, different concentrations of estrogen along with fixed concentration of insulin and different concentrations of progesterone along with fixed concentration of insulin as indicated for 4 h at 37°C under sterile conditions, and when needed the nucleic acids were isolated from these incubated samples for *in vitro* translation of maspin as described below.

### Separation of plant ribosome

Bael leaves (*Aegle marmelos*) was taken and washed thoroughly. It is rinsed using distilled water and homogenized. After centrifugation at 5000 rpm to remove the debris, the supernatant was taken. The supernatant was layered on the top of a 1 nM sucrose cushion and again centrifuged at 200,000 g to get the pellet of ribosomes (8).

### *In vitro* translation of maspin–mRNA

Nucleic acids containing maspin mRNAs were isolated by Trizol methods from the neutrophils isolated from blood samples from breast cancer patients and from the normal volunteers (14). The nucleic acid preparation was incubated with ribosomal preparation, mixture of all amino acids (0.1 μmol each/ml) and 2 mM ATP as described (15). After 6 h of incubation under sterile condition the reaction mixture at 0°C was centrifuged at 10,000 g for 10 min. The supernatant was used for the determination of maspin by ELISA as described below.

### Enzyme linked immunosorbant assay (ELISA) for Maspin

Maspin was quantitated by ELISA using polyclonal antibody developed against rh Maspin (8). ELISA was performed by the method as described before (16).

### Scatchard plot analysis of the equilibrium binding of estrogen and progesterone to their receptors in neutrophils

The neutrophil suspensions were prepared from the blood samples from normal and suspended (6 × 10^9^ cells/L) in HBBS buffer, pH7.4, with different amounts of pure estrogen and progesterone and incubated for different periods of time at 37°C. After incubation, the neutrophils with the bound estrogen and progesterone were separated from the unbound hormones by filtration over glass microfibre filter (GF/C, Sigma Chemicals Co.) using milipore filter as described before (6). After the filtration, the neutrophils were washed two times with equal vol of the buffer. The GF/C filter that retained the neutrophils with the bound hormones was subsequently air dried and estrogen and progesterone were eluted from the filter by trituration with 1 ml of CHCl_3_-CH_3_OH (1:1) mixture. After centrifugation at 0°C at 5000 g, portions of the supernatant were air dried. The air dried samples were redissolved in 0.9% NaCl and the concentrations of estrogen and progesterone in the sample were determined by ELISA. The results obtained were verified further by using 1.0 μci 4-^14^C –Estrogen, 1.0 μci 4-^14^C –Progesterone (JTJADEN, Bioscience) to the incubation mixture. The bound estrogen and progesterone were separated from the unbound ligands as described above and the radioactivity was measured to determine the binding in a scintillation counter as described (6).

The specific estrogen and progesterone binding were determined by adding 10 mM unlabelled estrogen and progesterone to the radio labeled estradiol and progesterone as described above after subtracting the nonspecific binding from the total binding. The dissociation constant (Kd) and the receptor numbers (n) from Scatchard plots (17) were determined by computer analysis.

### Equilibrium binding of ^125^I- insulin to neutrophils for the construction of scatchard plot

Porcine ^125^I-insulin was prepared and purified before the radioiodinated insulin was used for the binding assay as described before (18). The desired amounts of insulin were made by mixing the ^125^I-insulin with similarly purified unlabelled porcine insulin. The binding of insulin to the neutrophils was studied by filtration on GF/C membrane filtration technique using Milipore filter as described before (6). The nonspecific binding of insulin to the neutrophils was studied by mixing 0.7 μM unlabelled insulin with ^125^I-insulin.

The binding characteristics of insulin to the neutrophils were analysed by scatchard plot (17).

### Statistical analyses

The results obtained are presented as mean ± SD (standard deviation), and the significance of the results was determined by Students’t-test, and *p*<0.005 was considered to be significant. GraphPad Prism is the software used for statistical analyses.

## RESULTS

### Scatchard plot of the equilibrium binding of insulin to intact normal neutrophils

**Figure 1:**
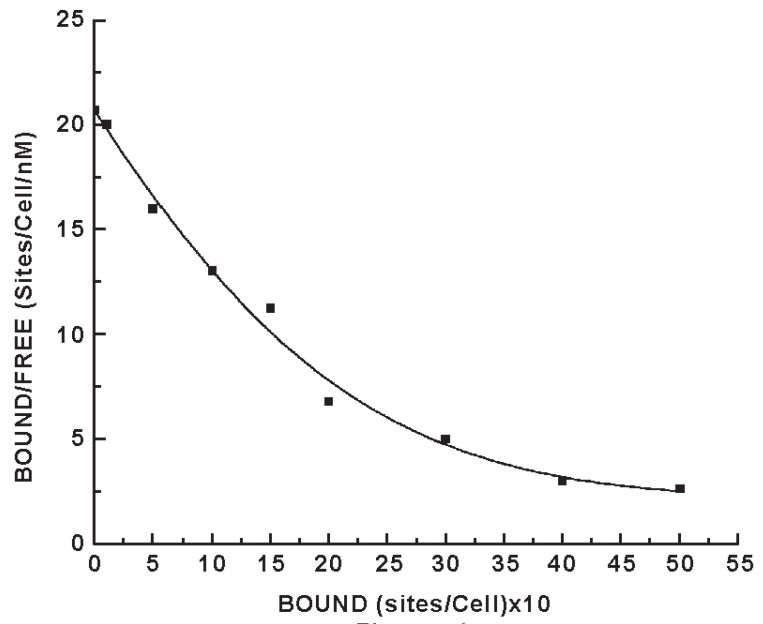
Scatchard plot of the equilibrium binding of insulin to normal neutrophils. The equilibrium binding of insulin to neutrophils was carried out by incubating purified porcine ^125^I-insulin in the binding assay mixture as described in the Materials and Methods. After incubation for 2.5h at 23°C, the bound ligand was separated from the free insulin by using glass micro fibre filter (GF/C) in a Millipore manifold filtration unit as described (6).

The incubation of insulin to the neutrophils also demonstrated highly specific insulin binding sites to neutrophils (19, 20). Scatchard plot of the binding of insulin to neutrophils, however produces typical curvilinear profile of equilibrium binding of the hormone to neutrophils (Figure 1). The insulin binding profile indicated heterogeneous insulin receptor populations. The analysis of the binding profile demonstrated the presence of one high affinity (Kd_1_) low capacity (n_1_) receptor binding population and one low affinity (Kd_2_) high capacity insulin receptor population (n_2_) on the neutrophils. However it is generally accepted that it’s the high affinity (Kd_1_ = 1.1 × 10^-9^ M), low capacity (n_1_ = 340 ± 20 binding sites/cells) insulin binding site are physiologically more important for the insulin effect than that of the other receptors population on the cell surface (i.e. Kd_2_, n_2_) of insulin. The low affinity receptors with high capacity binding are considered to be nonspecific binding. For this reason high affinity, low capacity insulin receptors population in the neutrophils was considered to be relevant to our study.

### Scatchard plot of the equilibrium binding of estrogen to intact normal neutrophils

**Figure 2:**
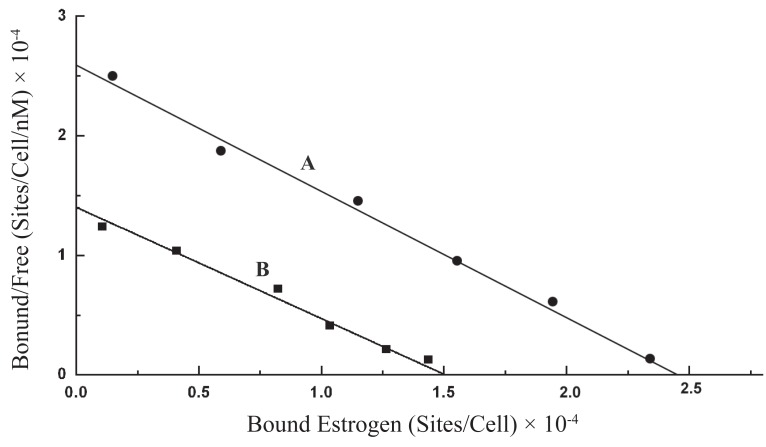
Scatchard plot of estrogen binding to normal neutrophils pre incubated with or without insulin. A. Scatchard plot of equilibrium estrogen binding to normal neutrophils; B. Scatchard plot of equilibrium estrogen binding to the normal neutrophils pre incubated with insulin. Detail of the equilibrium binding of estrogen to neutrophils was carried out as described in Materials and Methods. The estrogen was quantitated by ELISA using polyclonal antibody raised in rabbits as described in the Materials and Methods. In the experiment the neutrophils were treated with 200 μunits of insulin for 2.5 h at 23°C before these cells were used for the study of binding of estrogen without removing insulin from the cell suspension.

Scatchard plot of the equilibrium binding of estrogen to neutrophils demonstrated typical homogeneous estrogen receptor population (Figure 2A). The analysis of the binding characteristics showed there were 4.179 ± 1.02 × 10^7^ estrogen receptor binding sites/cell, with dissociation constant (Kd) 0.926 nM.

### Scatchard plot of the equilibrium binding of progesterone to intact normal neutrophils

**Figure 3:**
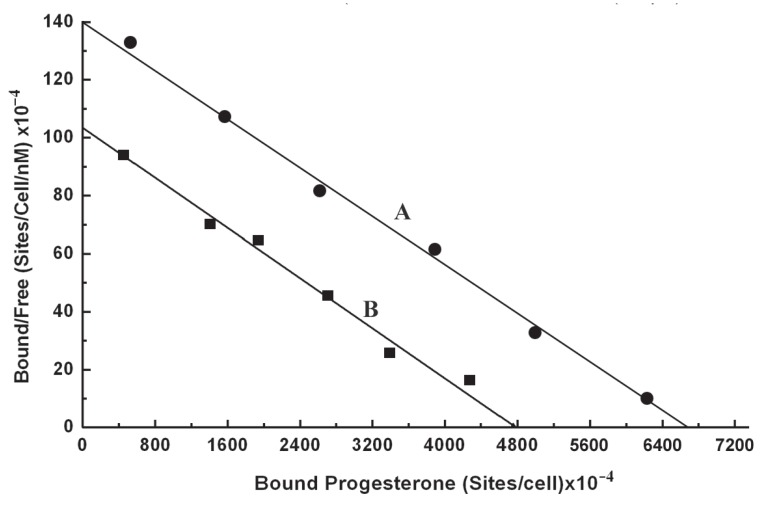
Scatchard plot of the equilibrium binding of progesterone to normal neutrophils pre incubated with or without insulin. The equilibrium binding of progesterone to the normal neutrophils was carried out by incubating these cells with progesterone as described in the Materials and Methods. The progesterone binding was determined by ELISA using polyclonal antibody raised in rabbits. A, Scatchard plot of the equilibrium binding of progesterone to neutrophils incubated in the absence of insulin; B, Scatchard plot of the equilibrium binding of progesterone to the normal neutrophils preincubated with insulin. Neutrophils to suspension were incubated with 200 μU of insulin for 2.5 h at 23°C to reach equilibrium binding of insulin to these cells. These cells were next treated with progesterone to determine the binding of progesterone to neutrophils pre incubated with insulin without removing insulin from the binding assay mixture.

As in the case of estrogen the equilibrium binding of progesterone to normal neutrophils, also presented a typical linear scatchard plot (Figure 3A). The plot indicated the homogeneous progesterone receptor sites on normal neutrophils membrane surface. The analysis of scatchard plot demonstrated the dissociation constant (Kd) = 47.619 nM with 11.5 × 10^10^ binding sites of progesterone/ cell.

### Effect of pre incubation of neutrophils with insulin on the binding of estrogen to its receptors on these cells

Neutrophils were isolated from the peripheral blood of normal volunteers and the cells were incubated for 2.5 h at 23°C with 200 μU of porcine insulin to reach equilibrium as described in the Materials and Methods. The neutrophils that were pre incubated with insulin were next reacted with estrogen to determine the binding of estrogen to these cells. Scatchard plot of the equilibrium binding of estrogen to the neutrophils pre incubated with insulin was constructed and compared with that constructed using neutrophils that were not pre incubated with insulin (Figure 2B). It was found that as a result of incubation of neutrophils with insulin the binding affinity for estrogen to its receptors in neutrophils remained essentially unchanged which demonstrated Kd = 1.072 nM compared to Kd of the binding of estrogen is 0.926 nM in the neutrophils that were not pretreated with insulin. The estrogen receptors which were 4.179 × 10^7^ /cell in the untreated cells was found to be decreased to 2.586 × 10^7^/cell (*p*<0.005, n=6) after the same cell were treated with 200 μU of insulin. These results demonstrated that as a result of pre treatment of neutrophils with insulin, the estrogen binding sites in the neutrophils were found to be decreased by >38% when compared to that in the control experiment.

### Effect of treatment of neutrophils with insulin on the binding of progesterone to its receptors

In separate experiments the normal neutrophils were isolated and incubated with 200 μU/ml insulin for 2.5 h at 23°C to achieve the equilibrium binding of insulin to these cells. These neutrophils pre incubated with insulin were subsequently used to determine the binding of progesterone, and the equilibrium binding of progesterone to these cells was analysed by scatchard plot. Scatchard plot for binding of progesterone to the neutrophils was compared with scatchard plot of the binding of progesterone to the neutrophils that were pre incubated with insulin (Figure 3B). It was found that Kd of the binding of progesterone to normal neutrophils which was 47.619 nM with progesterone receptor number of 11.5 × 10^10^/cell. The same neutrophils were incubated with insulin and the insulin treated neutrophils were used to determine the binding of progesterone. When the binding characteristics of progesterone was analysed by scatchard plot, it was found that while the Kd of progesterone binding to neutrophils compared to normal neutrophils remained essentially unchanged (Kd = 46.08 nM) due to the treatment of the cells with insulin, the number of progesterone binding sites however in the insulin treated neutrophils was found to be decreased to 8.2 × 10^10^/cell (*p*<0.005, n=6). This result indicated a 27% decrease of progesterone receptor number on the neutrophils due to the pretreatment of these cells to insulin without altering the dissociation constant of the progesterone binding to its receptor sites in the neutrophils.

### The effect of binding of estrogen to neutrophils pre incubated with insulin on the nitric oxide induced synthesis of maspin

**Figure 4:**
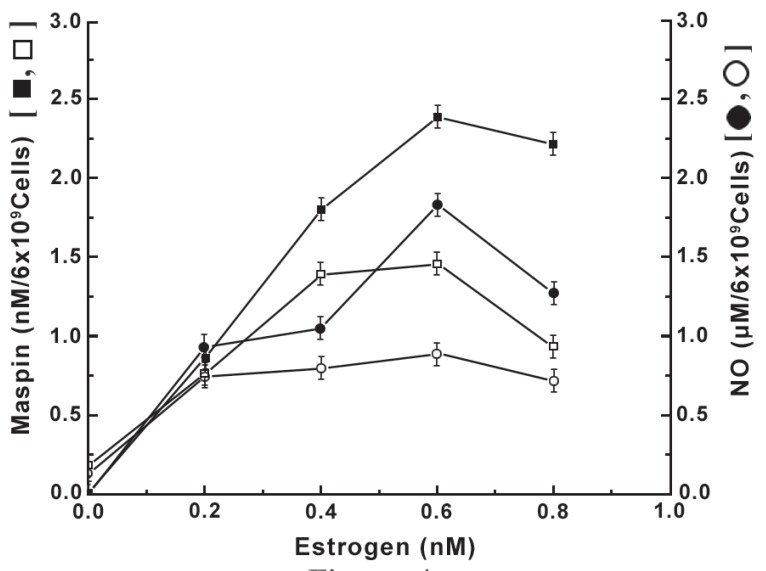
Effect of pre incubation of normal neutrophils with insulin on the estrogen induced NO and maspin synthesis. Normal neutrophils suspension was pre incubated with 200 μU of insulin for 2.5 h at 23°C to attain equilibrium binding of the hypoglycemic protein. The neutrophils pre incubated with insulin were next treated with different amounts of estrogen as indicated. After incubation for 4 h at 37°C the estrogen induced synthesis of both NO and maspin were subsequently determined. Solid square (▪) = effect of estrogen induced maspin synthesis; Hollow square (□) = effect of pre incubation of neutrophils with insulin on the estrogen induced maspin synthesis; Solid circle (●) = estrogen induced NO synthesis in neutrophils; Hollow circle (o) = estrogen induced NO synthesis in neutrophils pre incubated with insulin; Each point is mean ± S.D. of five different experiments each in triplicate using blood samples from 10 different donors.

It has been reported before that the treatment of neutrophils with estrogen led to the increased synthesis of maspin due to NO production as a result of interaction between the steroid and its receptors on the cell surface (7). As the manifestation of the estrogen effect was directly related to the estrogen receptor number on the cell surface, experiments were carried out to determine the role of insulin on the down regulation of estrogen receptor number on the neutrophil surface, on the steroid induced maspin synthesis through the stimulation of NO production (Figure 4). It was found that not only the synthesis of NO in the presence of different concentrations of estrogen was reduced in the neutrophils pre incubated with insulin, but the estrogen induced maspin synthesis was also reduced in all cases. It was also found that the degree of reduction of the maspin synthesis in the neutrophils pre incubated with insulin was directly related to the reduction of the amount of NO synthesis at all concentrations of estrogen used in the assay mixture.

### The effect of pre incubation of neutrophils with insulin on the progesterone induced maspin synthesis due to the stimulation of NO production

**Figure 5:**
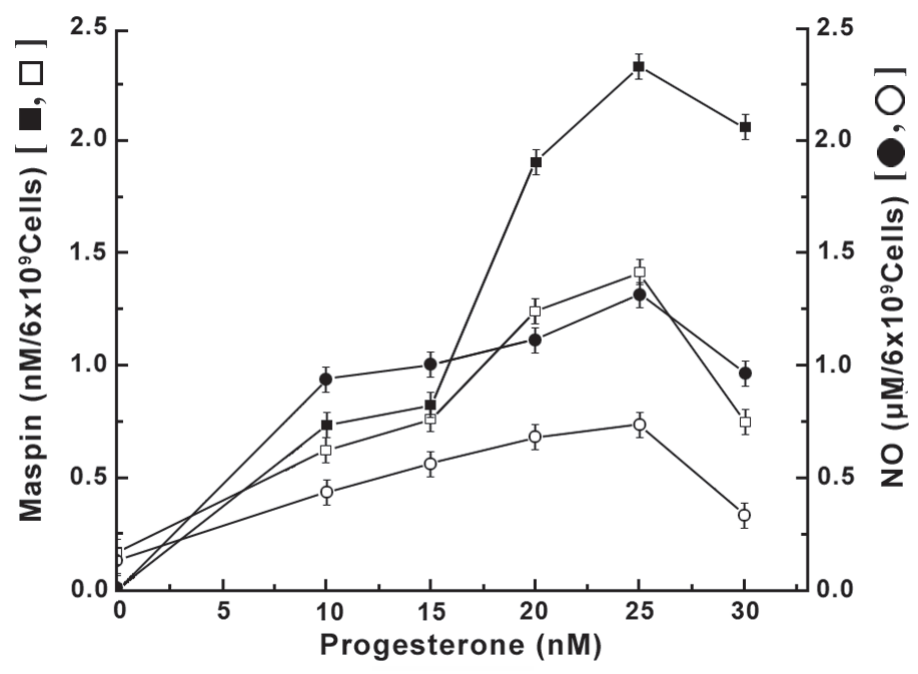
Effect of pre incubation of normal neutrophils with insulin on the progesterone induced synthesis of NO and maspin. Normal neutrophils suspension was incubated with 200 μU of insulin to reach equilibrium binding of the protein hormone as describing under Figure 4. These cells were next treated with different amounts of progesterone as indicated and after incubation for 4 h at 37°C, the synthesis of both NO and maspin was determined. Solid square (▪) = progesterone induced maspin synthesis in neutrophils; Hollow square (□) = progesterone induced maspin synthesis in neutrophils pre incubated with insulin; Solid circle (●) = progesterone induced NO synthesis in neutrophils; Hollow circle (o) = progesterone induced NO synthesis in neutrophils previously incubated with insulin for 2 h at 23°C; Each point is mean±S.D.of five different experiments each in triplicate using blood samples from 10 different donors.

As in the case of estrogen, the treatment of normal neutrophils with progesterone, which is currently believed to oppose the estrogenic effects (21), resulted in the increased synthesis of maspin through the increased NO production at least in normal neutrophils (Figure 5). However it was also found that the estrogen was ≈41 fold more potent inducer of maspin synthesis, that associated with NO production compared to those produced by progesterone as described in Figure 4. On the other hand, the treatment of neutrophils that were pre incubated with insulin was found to down regulate the progesterone receptor number on the cell surface (Figure 3B) resulted in the reduction of both NO and maspin synthesis at different concentrations of progesterone. And in each case the reduced amount of NO production was found to be directly related to the reduction of progesterone induced maspin synthesis (Figure 5).

### The effect of estrogen on the synthesis of NO and maspin in ER+ and ER– neutrophils pre incubated with insulin

As the above results indicated that the pre incubation of normal neutrophils with insulin led to the impaired synthesis of both NO and maspin (Figure 4), the effect of estrogen on the synthesis of NO and maspin in ER+ and ER– neutrophils, that were pre incubated with insulin was determined. It was found that the pre incubation of ER+ neutrophils with insulin resulted in the significant impairment of the synthesis of both NO and maspin in these cells when compared to the control (Table 1). The treatment of ER– neutrophils which failed to produce either NO or maspin when treated with estrogen also failed to produce these agents when these cells were pretreated with insulin.

**Table 1:** The effect of estrogen induced NO and maspin synthesis in ER+ and ER– neutrophils pretreated with or without insulin

Addition	Maspin (nM/6 × 10^9^ cells)		NO (μM/ 6 × 10^9^ cells)	
	ER+	ER–	ER+	ER–

Estrogen (0.6 nM) cells not pre incubated with insulin	1.422 ± 0.029^a^	0	0.887 ± 0.003^b^	0
Estrogen (0.6 nM) + Insulin (200 μU) cells pre incubated with insulin	0.790 ± 0.004^a^	0	0.470 ± 0.003^b^	0

Estrogen receptor positive (ER+) and estrogen receptor negative (ER–) neutrophils isolated from ER+ and ER– breast cancer subjects respectively were suspended in HBBS buffer and treated with 0.6 nM estrogen for 4 h at 37°C. After incubation estrogen induced NO and maspin syntheses in these cells were determined. In parallel experiment both ER+ neutrophils and ER– neutrophils were pre incubated with 200 μunits of insulin for 2.5 h at 23°C to allow the attainment of equilibrium binding of insulin to these cells. These pre incubated neutrophils next were treated with 0.6 nM estrogen for 4 h at 37°C and estrogen induced NO and maspin synthesis in these cells preparation was determined.

a *P*<0.005, n=10;

b *P*<0.001, n=10.

### The effect of progesterone on the synthesis of NO and maspin in PR+ and PR– neutrophils pretreated with insulin

As in the case of estrogen on the synthesis of NO and maspin in ER+ neutrophils pretreated with insulin that resulted in the estrogen induced production of both NO and maspin, when PR+ and PR– neutrophils were incubated with insulin to reach equilibrium binding and subsequently treated with progesterone, the production of both NO and maspin were also significantly impaired when compared to the PR+ neutrophils not treated with insulin (Table 2). The incubation of PR– neutrophils in the presence or absence of insulin produced no effect on the steroid induced synthesis of NO or maspin.

**Table 2:** The effect of progesterone on the synthesis of NO and maspin in PR+ and PR– neutrophils pre incubated with or without insulin

Addition	Maspin (nM/ 6 × 10^9^ cells)		NO (μM/ 6 × 10^9^ cells)	
	PR+	PR–	PR+	PR–

Progesterone (25 nM) cells not pre incubated with insulin	1.138 ± 0.024^a^	0	0.720 ± 0.006^b^	0
Progesterone (25 nM) + Insulin (200 μU) cells pre incubated with insulin	0.555 ± 0.003^a^	0	0.313 ± 0.002^b^	0

Progesterone receptor positive (PR+) and progesterone receptor negative (PR–) neutrophils were isolated from PR+ and PR– breast cancer subjects. The effect of progesterone (25 nM) on the synthesis of NO and maspin was determined after 4 h of incubation with progesterone at 37°C. In parallel experiment PR+ and PR– neutrophils were treated with 200 μunits of insulin for 2.5 h at 23°C. After incubation 25 nM progesterone was added to the reaction mixture. After the second incubation for 4 h at 37°C, the production of NO and maspin were determined.

a *P*<0.005, n=10;

b *P*<0.001, n=10.

### Effect of estrogen, nitric oxide (NO), and NAME on maspin synthesis in normal, estrogen receptor + (ER+), and ER– neutrophils

Treatment of normal and ER+ neutrophils with 5 μM NO (final) solution in 0.9% NaCl, instead of estrogen, produced 2.90 ± 0.17 nM and 1.95 ± 0.13 nM maspin (*p*<0.005, n=5) respectively. Interestingly, although the treatment of ER– neutrophils with estrogen failed to produce any NO or maspin, the addition of NO solution to the ER– cells resulted in the synthesis of 0.87 ± 0.005 nM maspin even in the absence of added estrogen to the incubation mixture (*p*<0.005, n=5). The addition of NAME to the reaction mixture containing NO for the synthesis of maspin had no effect on the synthesis of the anti - breast cancer protein. These results indicated that although NO was capable of inducing maspin synthesis in ER– neutrophils, the addition of NAME, an inhibitor of nitric oxide synthase, had no effect on NO or the synthesis of maspin in the presence of added NO. In other words, NAME had no effect on maspin synthesis induced by added NO to the reaction mixture containing neutrophils (Table 3).

**Table 3:** Effect of estrogen, nitric oxide (NO), and NAME on maspin synthesis in normal, estrogen receptor + (ER+), and ER– neutrophils

Maspin(nM/6 × 10^9^ cells)			
	NORMAL	ER+	ER-

Estrogen (0.6 nM)	2.383 ± 0.014^a^	1.422 ± 0.029^b^	0
NO (5 μM)	2.90 ± 0.17^a^^c^	1.95 ± 0.13^b^^c^	0.870 ± 0.005^c^
Estrogen (0.6 nM) + NAME (10 mM)	0	0	0
NO (5 μM) + NAME (10 mM)	2.90 ± 0.17	1.95 ± 0.13	0.870 ± 0.005

The neutrophil suspensions were prepared from the blood of patients with ER+ or ER– breast cancer and of age-matched normal female volunteers as described in the Materials and Methods section. The neutrophil preparations (6 × 10^9^ cells/L) were suspended in Hank’s balanced salt solution (pH7.4) and treated with estrogen (0.6 nM) and NO (5 μM). In a separate experiment, a neutrophil suspension was incubated with NAME (10 mM) and with either NO or estrogen. Results are mean ± SD of five different experiments in triplicate using blood of 15 patients with breast cancer and 15 normal female volunteers.

a *P*<0.005, n=5;

b *P*<0.005, n=5;

c *P*<0.005, n=5.

### Effect of progesterone, nitric oxide (NO), and NAME on maspin synthesis in normal, progesterone receptor + (PR+), and PR– neutrophils

Treatment of normal and PR+ neutrophils with 5 μM NO (final) solution in 0.9% NaCl, instead of progesterone, produced 2.752 ± 0.15 nM and 1.821 ± 0.16 nM maspin (*p*<0.005, n=5) respectively. Interestingly, although the treatment of PR– neutrophils with progesterone failed to produce any NO or maspin, the addition of NO solution to the PR– cells resulted in the synthesis of 0.692 ± 0.008 nM maspin even in the absence of added progesterone to the incubation mixture (*p*<0.005, n=5). The addition of NAME to the reaction mixture containing NO for the synthesis of maspin had no effect on the synthesis of the anti-breast cancer protein. These results indicated that although NO was capable of inducing maspin synthesis in PR– neutrophils, the addition of NAME, an inhibitor of nitric oxide synthase, had no effect on NO or the synthesis of maspin in the presence of added NO.

In other words, NAME had no effect on maspin synthesis induced by added NO to the reaction mixture containing neutrophils (Table 4).

**Table 4:** Effect of progesterone, nitric oxide (NO), and NAME on maspin synthesis in normal, progesterone receptor + (PR+), and PR– neutrophils

Maspin(nM/6 × 10^9^ cells)			
	NORMAL	PR+	PR-

Progesterone (25 nM)	2.329 ± 0.012^a^	1.138 ± 0.024^b^	0
NO (5 μM)	2.752 ± 0.15^a^^c^	1.821 ± 0.16^b^^c^	0.692 ± 0.008^c^
Progesterone (25 nM) + NAME (10 mM)	0	0	0
NO (5 μM) + NAME (10 mM)	2.752 ± 0.15	1.821 ± 0.16	0.692 ± 0.008

The neutrophil suspensions were prepared from the blood of patients with PR+ or PR– breast cancer and of age-matched normal female volunteers as described in the Materials and Methods section. The neutrophil preparations (6 × 10^9^ cells/L) were suspended in Hank’s balanced salt solution (pH7.4) and treated with progesterone (25 nM) and, NO (5 μM). In a separate experiment, a neutrophil suspension was incubated with NAME (10 mM) and with either NO or progesterone. Results are mean ± SD of five different experiments in triplicate using blood of 15 patients with breast cancer and 15 normal female volunteers.

a *P*<0.005, n=5;

b *P*<0.005, n=5;

c *P*<0.005, n=5.

## DISCUSSION

Hormones in general are well known for their specific effects in the target cells through their specific binding to the cell surface membrane. As the interaction of the hormone with its receptor site ultimately determines the expression of the hormone effect, the ‘cross talk’ between the hormone receptors may significantly either enhance or impair the activity of unrelated hormones in the system. In the case of insulin which has its own specific receptors for its transduction of its hypoglycemic effects in target cells for glucose metabolism. It was however found that the hypoglycemic hormone influenced the activity of both estrogen and progesterone which are known to induce the synthesis of maspin, an anti breast cancer protein through the stimulation of NO synthesis in normal as well as ER+ and PR+ neutrophils from the breast cancer patients (Table 1 and Table 2). Both ER– and PR– reported to produce worse prognostic outcome of the disease when compared to that in ER+ and PR+ breast cancer patients (10, 22-24). And in this sense our results suggested that the well known anti diabetic protein might bring forth un towards effect through the impairment of the synthesis of maspin, which reported to possess several anti breast cancer properties including anti metastatic, anti invasive effect against breast cancer (25, 26).

It has also been reported that ER+ neutrophils from the breast cancer patients produced less amount of NO induced maspin synthesis compared to normal control (7). As reported above the amount of progesterone induced maspin production was also impaired in PR+ neutrophils compared to normal neutrophils.

However the pre incubation of either ER+ neutrophils or PR+ neutrophils with insulin resulted in the down regulation of both estrogen and progesterone receptor numbers in the intact neutrophils that resulted in the impairment of NO induced maspin production by these steroid hormones. The down regulation of hormone receptor numbers by the same hormone receptor interaction, usually called “homologous” receptor down regulation is known in the case of insulin for same, where the binding of one molecule of the ligand to its receptor is reported to cause decrease of the binding of a second molecule of insulin to its receptor, generally known as the negative co operatively among the receptor which could be of physiologically important in the prevention of insulin induced hypoglycemia (27). In contrast to the homologous down regulation, the existence of heterologous up and down regulation of the receptor number by different hormone or prostaglandins are also known. For example while prostacyclin is known to up regulate insulin receptor number in erythrocyte membrane prostaglandin E_2_ is reported to down regulate insulin receptor number in the same membrane (4). It should also be mentioned here that the heterologous up regulation or down regulation i.e. “crosstalk” may involve the stimulation or inhibition of hormone receptor (protein) synthesis, but crosstalk has reported to be independent of protein synthesis and the actual number of receptors on the cell surface was due to the exposure of the spare receptor in the membrane bilayer (6). The effect of this hypoglycemic hormone, as described in our study represented a case of heterologous down regulation of both estrogen and progesterone receptors in neutrophils mediated through an apparent “cross talk” between different receptors. Those results implied that the systemic presence of insulin might adversely affect the systemic production of the anti breast cancer protein. However the insulin induced down regulation of the steroid receptor numbers in the neutrophils that not only resulted in the estrogen or progesterone induced maspin synthesis, but also inhibited NO synthesis induced by the steroids in these cells. As increased NO synthesis has been reported to induce the synthesis of interferon α which has also been reported to produce anti breast cancer activities (28, 29). And, as such, the down regulation of estrogen or progesterone receptor number by insulin in neutrophils under normal or under the pathologic condition of breast cancer might result in the impairment of systemic synthesis of interferon α which is also reported to be synthesized in these cells in the presence of NO (30).

It might also be speculated that the hyperinsulinemia in type2 diabetes mellitus or due to the excessive use of insulin to control hyperglycemia in diabetes mellitus might influence the development of breast cancer by impairing the systemic synthesis of maspin. As insulin was found to down regulate maspin synthesis, it could perhaps be speculated the occurrence of type II diabetes mellitus that is reported to cause hyper insulinemia due to systemic insulin resistance might actually lead to worse prognostic outcome in breast cancer in patients with co occurrence of type II diabetes mellitus. Fallaciously, the occurrence of type I diabetes mellitus where insulin synthesis is known to be completely impaired might be beneficial in breast cancer due to maspin synthesis. On the other hand interferon α which are reported to have antibreast cancer activities (25, 26, 28, 29) through the “cross talk” between the receptors of insulin, estrogen and progesterone.
